# The Fatigue and Altered Cognition Scale among SARS-CoV-2 Survivors: Psychometric Properties and Item Correlations with Depression and Anxiety Symptoms

**DOI:** 10.3390/jcm13082186

**Published:** 2024-04-10

**Authors:** Yu-Yu Hsiao, Timothy R. Elliott, Julie Jaramillo, Megan E. Douglas, Mark B. Powers, Ann Marie Warren

**Affiliations:** 1 Department of Individual, Family and Community Education, University of New Mexico, Albuquerque, NM 87131, USA; yuyuhsiao@unm.edu (Y.-Y.H.); jjaramillo8@unm.edu (J.J.); 2 Department of Educational Psychology, Texas A&M University, College Station, TX 77843, USA; 3 Baylor Scott & White Research Institute, Dallas, TX 75246, USA; megan.e.douglas2.civ@health.mil (M.E.D.); mark.powers1@bswhealth.org (M.B.P.); annmarie.warren@bswhealth.org (A.M.W.)

**Keywords:** brain fog, fatigue, assessment, SARS-CoV-2 infection, cognition

## Abstract

**Background/Objectives**: This study examined the psychometric properties of the Fatigue and Altered Cognition Scale (FACs) among adult COVID-19 survivors and its unique ability to assess symptomology not accounted for by measures of depression and anxiety. **Methods**: COVID-19 survivors completed an online survey that included the FACs, a measure of brain fog and central fatigue with 20 items rated on a digital–analog scale. Useable data from 559 participants were analyzed to test the two-factor structure of the FACs, test for measurement invariance by sex and device was used to complete the survey (hand-held, computer), and item correlations with symptoms of depression and anxiety were examined. **Results**: The two-factor structure of the FACs replicated, supporting the separate assessments of brain fog and fatigue, $\chi^{2}$(164) = 1028.363, *p* < 0.001, CFI = 0.934, TLI = 0.923, RMSEA = 0.097, SRMR = 0.053. The FACs exhibited invariance at the scalar level, indicating item and factor integrity regardless of sex and device type. Using a correlation > 0.70 as a criterion (i.e., indicating more than 50% shared variance between two items), items on the FACs (assessing fatigue and lack of energy) were highly correlated with feeling tired or having little energy on the depression measure. No other items correlated with any anxiety symptom larger than 0.70. **Conclusions**: The FACs appears to be a psychometrically sound and efficient measure for use with COVID-19 survivors, assessing symptoms of brain fog and central fatigue that are not attributable to symptoms assessed by established measures of depression and anxiety.

## 1. Introduction

Brain fog and persistent fatigue appear to be cardinal symptoms of post-acute sequelae of SARS-CoV-2 infection (PASC; [1,2]). Other neuropsychiatric problems associated with PASC include sleep difficulties, anxiety, depression, post-traumatic stress, lack of interest or pleasure in usual activities, malaise, and cognitive dysfunctions such as indecision, confusion, and difficulties with attention and concentration [3]. A recent review of this literature concluded that approximately 90% of patients hospitalized for COVID-19 report at least one neuropsychiatric symptom six months post-discharge, and at least one is reported by approximately 25% of non-hospitalized COVID-19 survivors [4].

Our clinical understanding of these neuropsychiatric symptoms—specifically brain fog and fatigue—is undermined by definitional and methodological issues. These issues have direct consequences on clinical assessment, treatment planning, and symptom monitoring. There is no professional consensus in defining or measuring brain fog, for example. This colloquial term is used by clinicians and patients to describe an array of subjective problems with mental clarity, forgetfulness, concentration, and attention, which are indicative of issues with short-term and working memory, and processing speed [5,6]. Similarly, operational definitions of fatigue are rare, ignoring important distinctions between *peripheral* and *central* fatigue. Most studies do not specify the kind of fatigue under investigation, deferring to the definitions associated with the self-reported measures of fatigue that are used [7]. Muscular exhaustion or impairment due to exertion describes peripheral fatigue, and central fatigue is a subjective report of difficulties initiating and maintaining goal-directed activity, particularly when sustained mental effort is required [8].

Imprecise definitions of brain fog and central fatigue and their symptoms conflate these conditions with other neuropsychiatric disorders. The fifth and revised edition of the Diagnostic and Statistical Manual of Mental Disorders [9] stipulates that to diagnose a major depressive disorder, a person must report either depressed mood or a significant loss of interest or pleasure in activities over a two-week period, and at least four of the following symptoms that occur nearly every day during that time frame: fatigue or decreased energy, psychomotor retardation or agitation, sleep disturbance, feelings of worthlessness or inappropriate guilt, recurrent thoughts of death or suicidal ideation, and difficulties thinking, concentrating, or being indecisive during the same time frame (and these symptoms are not attributable to an existing physical condition). To diagnose a generalized anxiety disorder, a person must report excessive worry or anxiety most of the time for the previous six months (Criterion A), difficulty controlling the worry and/or anxiety (Criterion B), and at least three other co-occurring symptoms that include fatigue, sleep disturbance and difficulty concentrating (and are not attributable to an existing medical condition). Most studies reporting significant associations between brain fog and fatigue with depression and anxiety rely on the total scores or cut-off scores from self-report measures of depression and anxiety and fail to examine specific symptom patterns [10]. Consequently, we do not yet understand the degree of overlap between features commonly ascribed to brain fog and central fatigue with symptoms specific to clinical syndromes of depression and anxiety.

Understanding the overlap between symptoms of depression and anxiety with brain fog associated with PASC, in particular, is compromised by the lack of operational definitions and corresponding assessments of brain fog. Various measures of cognitive dysfunction have been used to measure brain fog including neurobehavioral symptom checklists [11], brief cognitive screening devices [12], and comprehensive neuropsychological batteries that include multiple instruments [5,13]. Measures devised to screen for dementia may be insensitive to the symptoms associated with brain fog [14], and neuropsychological batteries are expensive, time consuming, and cumbersome, and may pose problems for patients with central fatigue. Furthermore, these instruments may be insensitive to a patient’s response to treatment [15].

In the present study, we examine the psychometric properties of Fatigue and Altered Cognition (FACs) [16] among COVID-19 survivors. The FACs was developed to efficiently assess symptoms of co-occurring brain fog and central fatigue among persons with chronic health conditions and its factor structure and item integrity were demonstrated among respondents with and without traumatic brain injury (TBI) [16]. The items on the FACs are formatted with digital visual analog response scales that can be administered with electronic devices in clinical and research settings. These features expedite its use in clinical assessment, treatment, and symptom monitoring. However, the two-factor structure of the FACs needs to be tested among COVID-19 survivors to determine its potential use as a clinical tool in assessing and monitoring co-occurring brain fog and fatigue associated with PASC. Further, studying the relationships between the symptoms of brain fog and fatigue with the specific symptoms of depression and anxiety could potentially elucidate our understanding of the degree to which these conditions overlap, considering the lack of research on this matter.

Co-occurring brain fog and fatigue occur across many chronic health conditions, including chronic fatigue syndrome [6], postural tachycardia syndrome [17], hypoparathyroidism [18], and fibromyalgia and rheumatoid arthritis [19]. This symptom complex is well-documented among persons with TBI [20,21], particularly among those who experience deficiencies in growth hormone (GH) and other pituitary secretions that frequently occur post-TBI [22,23]. GH and pituitary dysfunction appear to be a common pathway that contributes to the co-occurrence of brain fog and central fatigue across various chronic health conditions, including TBI and PASC [24]. Individuals with PASC also exhibit significantly lower GH secretions in comparison to those without PASC, and they report significantly more problems with fatigue, sleep, quality of life, and depression [24]. Preliminary evidence suggests that brain fog and fatigue among persons with TBI respond positively to GH replacement therapy [25,26]. Potentially, the FACs could be used to monitor patient response to treatments for brain fog and fatigue associated with PASC. Nevertheless, the psychometric properties of the FACs must be established among COVID-19 survivors to ensure its suitability for clinical and research purposes.

We conducted the present study to examine the psychometric properties of the FACs and item correlations with symptoms of depression and anxiety in an online survey of individuals who tested positive for COVID-19, and who agreed to participate in a longitudinal study of their psychological and behavioral experience of the pandemic. We tested the presumed two-factor structure of the FACs, and then conducted tests of measurement invariance by self-identified biological sex and by the type of device used to complete the survey (handheld devices and personal computers) to ensure the integrity of the items and the factor structure in the context of these potentially important variables. Finally, we conducted a series of correlational analyses to investigate the overlap between the items assessing brain fog and central fatigue on the FACs with symptoms of depression and anxiety assessed by two established, reliable, and valid measures.

## 2. Materials and Methods

### 2.1. Study Design

Individuals who tested positive for COVID-19 were invited to participate in an online “COVID-19 Digital Care Journey”, and informed consent was obtained electronically through the myBSW app. The survey was developed and managed using Research Electronic Data Capture (REDCap^TM^; [27,28]. REDCap is a secure, web-based electronic data platform hosted at Baylor Scott and White. The survey materials were only available in English, and recruitment occurred from 7 April 2020 to 19 April 2021. No compensation was provided for participating. The survey included several measures and took approximately 20 to 30 min to complete. Completed surveys were manually inspected for nonsensical open-text responses, duplicate responses, and odd patterns of missing data. All surveys were time-stamped, enabling inspection of completion times and dates. Complete details about recruitment methods and survey materials are described in Pogue et al. [29].

A total of 559 participants (average age 55.10 years, *SD* = 14.30) who answered “yes” to the question “Have you tested positive for the COVID-19 virus?” were selected as the study sample. Table 1 presents sample characteristics and demographic information. Fifty-eight participants reported hospitalization, and four had severe symptoms and were on ventilation while in the hospital. The average days of these patients stay in the hospital were 9.27 days (*SD* = 11.61). For patients who needed to be on ventilators, the average days they were on ventilators was 7.00 days (*SD* = 7.55). Most of the sample self-identified as female (*n* = 380, 67.98%), married (*n* = 382, 68.34%), with a bachelor’s degree or above (*n* = 364, 65.11%), and were employed (*n* = 335, 59.93%) at the time they took the survey.

### 2.2. Study Measures

The 20-item Fatigue and Altered Cognition Scale [16] contains 10 items to assess brain fog (labeled as “altered cognition”) and another 10 to assess central fatigue. Participants provided their responses to each item using an electronic visual analog rating (eVAS) scale, known for its simplicity in both administration and scoring [30]. Each item was anchored with extreme responses (“*not at all*” to “*extremely*”), and a “drag and drop” slider bar positioned in the middle of the horizontal rating scale was used by participants to indicate their responses. Each response was adjusted proportionally to achieve a score ranging from 0 to 100 for each item, aligning with current standards in the field [31]. Greater symptom severity is indicated by higher scores. Total scores are generated separately for brain fog and fatigue. The two scale scores of FACs have been shown to be reliable (α’s = 0.95) [16].

The Patient Health Questionnaire-8 (PHQ-8) [32] was used to assess symptoms of depression. The PHQ-8 consists of eight items that correspond to the diagnostic criteria for major depressive disorder as specified in the Diagnostic and Statistical Manual of Mental Disorders [9]. The PHQ-8 was used rather than the nine-item version due to the inability to safely monitor or assess suicidality in a large online survey. Items on the PHQ-8 are rated on a four-point Likert-type scale (that ranges from “*not at all*” to “*nearly every day*”). Higher scores represent a greater severity of symptoms. A total score > 9 is indicative of a probable major depressive disorder [33]. An acceptable internal consistency coefficient for the PHQ-8 scores was observed (α = 0.89).

The Generalized Anxiety Disorder Scale-7 (GAD-7) [34] was used to assess symptoms associated with generalized anxiety disorder. The scale includes questions about excessive worrying, restlessness, difficulty relaxing, and other common symptoms associated with generalized anxiety. Responses are scored on a four-point Likert scale from 0 (not at all) to 3 (nearly every day). Higher scores indicate greater severity of anxiety symptoms. A total score > 9 is indicative of a probable generalized anxiety disorder [34]. The internal consistency coefficient for the GAD-7 was acceptable (α = 0.94).

### 2.3. Analytic Plan

We first report several descriptive statistics at the item level for both central fatigue and altered cognition items, including missing response percentages, means, standard deviations, minimums, maximums, and skewness. These descriptive statistics were used to evaluate data and scaling qualities [35]. Next, the correlations between items and their corresponding scale scores were calculated. Item-scale correlations larger than 0.30 indicated the appropriateness of scoring items together on a single scale [35]. We estimated the reliability of the scale scores in the FACs. We calculated the reliability of the scale scores within the FACs. Internal consistency was assessed using Cronbach’s alpha [36], and the composite reliability values were calculated using the omega composite (Kline, 2020).

Confirmatory factor analysis (CFA) with maximum likelihood estimation was employed to determine the goodness of fit of a hypothesized model to the data. We specified the same two-factor model validated in Elliott et al. [16] on these COVID-19 patient data. The $\chi^{2}$ test and three local fit indices were used to determine how well the data fit the hypothesized model. The three local fit indices including the comparative fit index (CFI), the root mean square error of approximation (RMSEA), and the standardized root mean squared residual (SRMR) are commonly used to compensate for the $\chi^{2}$ test being overly sensitive to large sample size (*n* > 400) [37]. The suggested guidelines for adequate model fit indicate that RMSEA should be less than 0.10 [38,39,40], CFI > 0.90 [41], and SRMR < 0.10 [40,41].

Once the factor structure of the FACs was established, the measurement model of the FACs was further examined by performing the measurement invariance (MI) test by self-identified sex and then by device types used to complete the survey [42,43]. MI testing is a procedure to assess whether the measurement properties of the FACs construct are equivalent between groups. Multiple group CFAs were conducted to test a series of measurement models from the less restricted one to the most restricted one. The $\chi^{2}$ difference tests along with the differences in CFI, RMSEA, and SRMR between two nested models were adopted to evaluate whether the less restricted model outperforms the next more restricted one.

Last, Pearson’s *r* correlation coefficients between FACs items and both the PHQ-8 and GAD-7 items were estimated to examine the degree to which the FACs items overlap with symptoms of depression and anxiety. We anticipate the FACs items have some associations with both the PHQ-8 and GAD-7 items, given that items measuring fatigue and altered cognition share similar diagnostic aspects with depression and general anxiety (e.g., indecisiveness, problems concentrating, and loss of energy). However, we believe that FACs items also capture some unique aspects which are irrelevant to the PHQ-8 or GAD-7. Hence, we anticipated that the correlations should be smaller than 0.70, which indicates less than 50% of the shared variance between any FACs item with any depression or generalized anxiety item.

The descriptive statistics, Cronbach’s alpha coefficients, and Pearson’s *r* correlations were conducted using SPSS version 28 [44]. The omega composite reliability was calculated utilizing the R package MBESS [45]. CFA and measurement invariance tests were conducted using Mplus version 8.7 [46].

## 3. Results

### 3.1. Descriptive Differences

On the PHQ-8, 20.9% of participants had a total score of 10 or higher, suggesting a possible major depressive disorder, and 24.5% of participants had a total score of 10 or higher on the GAD-7, indicative of a possible generalized anxiety disorder. A total of 14.1% of the participants were above the clinical cut-off for both anxiety and depression. Women had a significantly higher average total score on the PHQ-8 (7.33, *SD* = 5.87) than men (4.84, *SD* = 5.31; *t* = 4.064, *p* < 0.001, Cohen’s *d* = 0.44). Similarly, women reported a significantly higher GAD-7 mean score (6.72; *SD* = 6.37) than men (4.01, *SD* = 5.51; *t* = 5.108, *p* < 0.001, Cohen’s *d* = 0.44). The average scores observed among women on both scales were in the mild range of severity [47].

Participants with clinically significant elevations on the PHQ-8 and the GAD-7 reported more problems with brain fog and fatigue, as measured by the FACs. Those with a possible major depressive disorder (i.e., PHQ-8 score > 9) exhibited, on average, fatigue scores of 74.69 (*SD* = 14.90), significantly higher than their non-depressed counterparts (*M* = 41.28, *SD* = 22.94; *t* = 17.58, *p* < 0.001, Cohen’s *d* = 1.59). Additionally, these depressive individuals had significantly higher altered cognition mean scores (64.12, *SD* = 20.68), than their non-depressive peers (*M* = 30.50, *SD* = 21.79; *t* = 14.73, *p* < 0.001, Cohen’s *d* = 1.56).

This pattern was also observed between participants who varied in their levels of anxiety. Respondents with scores exceeding the cut-off (GAD-7 > 9) reported significantly higher average fatigue (73.88, *SD* = 16.10) than those who had scores below this threshold (*M* = 42.23, *SD* = 23.73; *t* = 17.63, *p* < 0.001, Cohen’s *d* = 1.43). These individuals also reported significantly elevated altered cognition scores (65.72, *SD* = 20.27) than those who did not exceed the cut-off score (*M* = 31.97, *SD* = 22.89; *t* = 16.38, *p* < 0.001, Cohen’s *d* = 1.52).

### 3.2. Data Quality and Scaling Evaluation

Among the 559 patients, the amount of missing item-level data on the FACs ranged from 0.7% to 3.5%, which indicated high data quality [35]. The means across all items ranged from 28.44 to 58.98 and the standard deviations ranged from 24.37 to 33.13. Both the lowest rating of “0” and the highest rating of “100” were utilized by patients across all the 20 FACs items. These results indicated that the full spectrum of the rating scales was used by patients so the eVAS scale was appropriate for the FACs. The skewness estimates of all items were within the range of −1 to +1, indicating that the data followed a normal distribution [48]. Item-scale correlations ranged from 0.68 to 0.92, indicating strong correlations between FACs items scores and both the fatigue and altered cognition subscale scores.

### 3.3. Reliability

Cronbach’s alpha coefficients were high (fatigue, 0.96; altered cognition, 0.97). The omega reliability estimates for both the scores on the fatigue and altered cognition scale scores were 0.95. The robustness of both fatigue scale scores and cognition scale scores is reflected in these measures, implying that researchers can employ both scales to assess and compare individual differences with confidence [36].

### 3.4. Factor Structure

Following the procedure outlined in Elliott et al. [16], we conducted a comparison between the one-factor and two-factor models to assess their suitability in fitting the current data and gain insights into the underlying psychological construct among individuals affected by COVID-19. Model refinement was facilitated using modification indices [49]. Table 2 presents the descriptive statistics for the 20 items of the FACs.

The one-factor model exhibited inadequate fit to the data, as evidenced by $\chi^{2}$(170) = 2776.213, *p* < 0.001, CFI = 0.801, TLI = 0.777, RMSEA = 0.166, and SRMR = 0.060. On the other hand, the two-factor model exhibited some potential improvement in model fit indices, with $\chi^{2}$(169) = 1650.958, *p* < 0.001, CFI = 0.887, TLI = 0.873, RMSEA = 0.125, and SRMR = 0.060, suggesting an enhancement over the one-factor model in overall fit. The $\chi^{2}$ difference test between these two models revealed statistical significance, indicating that the two-factor model was significantly better than the one-factor model, $\chi^{2}$(1) = 1125.255 and *p* < 0.001. These findings offer preliminary support for the two-factor model of fatigue and brain fog.

**Figure 1:**
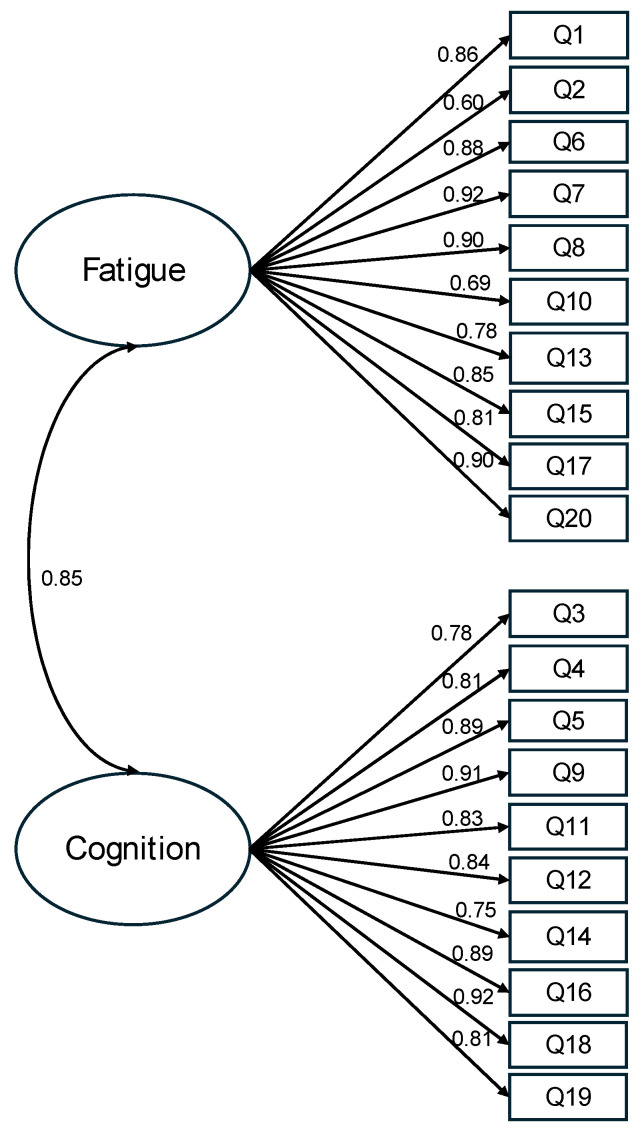
Standardized estimates of the two-factor model of the FACs. Note. The items’ residuals and the correlations of items residuals are omitted in this figure for clarity. This model has an acceptable fit, $\chi^{2}$(164) = 1028.363, *p* < 0.001, CFI = 0.934, TLI = 0.923, RMSEA = 0.097, and SRMR = 0.053.

After scrutinizing the modification indices, we introduced adjustments to enhance the model fit, specifically by incorporating correlations among the residuals of five item pairs (representing item variance unaccounted for by latent factors). These included correlations between item 4 (I was forgetful) and item 3 (I lost track of what I was going to say), between item 13 (I had to force myself to get things done) and item 17 (I had to struggle to finish what I started to do), between item 11 (I was easily confused) and item 12 (I felt “spaced out” like I was in a fog), between item 6 (I felt worn out) and item 8 (I felt run down), and between item 8 and item 7 (I felt sluggish). Following these adjustments, the revised model demonstrated a good fit to the data: $\chi^{2}$(164) = 1028.363, *p* < 0.001, CFI = 0.934, TLI = 0.923, RMSEA = 0.097, and SRMR = 0.053 (Figure 1). The correlation between the fatigue factor and the altered cognition factor was 0.85. With standardized factor loadings exceeding 0.70 for all but two items, it was evident that approximately 50% of the variance in the items was accounted for by the hypothesized factors. In summary, the results obtained from the confirmatory factor analysis support the two-factor model for depicting relationships among the FACs items among these COVID-19 survivors.

### 3.5. Measurement Invariance

Utilizing the two-factor model of FACs as a foundation, we proceeded to conduct subsequent tests for measurement invariance by self-identified sex groups (male; female) or the type of device used to complete the survey (handheld vs. computers). Initially, the configural invariance model was specified, where the same factor structure was estimated for both groups without imposing inter-group constraints on parameter estimates. Models established configural invariance would serve as the baseline models for metric invariance, wherein factor loadings were equated across groups. Satisfaction in metric invariance would lead to the examination of strong invariance with equal intercepts across groups. If the criteria for strong invariance between groups were met, we would proceed with a strong invariance test in which additional constraints of equal error variance across the two groups were applied. The $\chi^{2}$ difference test along with the fit indices criteria proposed by Chen [50] were adopted in evaluating various levels of invariance.

The results for measurement invariance across the two self-identified sex groups are presented in Table 3. All obtained goodness-of-fit indices met the criteria for configural invariance. Next, the criteria for goodness-of-fit indices for metric invariance between males and females were satisfied (Δ$\chi^{2}$(18) =22.06, *p* = 0.229, ΔRMSEA < 0.001, ΔCFI < 0.001, and ΔSRMR = 0.001). Furthermore, the requirements for goodness-of-fit indices for the scalar invariance test were met (Δ$\chi^{2}$(18) =27.98, *p* = 0.062, ΔRMSEA = 0.001, ΔCFI = −0.001, and ΔSRMR = 0.001). Subsequently, all criteria except for the *χ*^2^ difference test for strict in-variance indices were fulfilled ($\chi^{2}$(20) = 80.86, *p* < 0.001, ΔRMSEA = 0.001, ΔCFI = −0.005, and ΔSRMR = 0.002), thereby establishing strict invariance.

A second measurement invariance test was conducted to compare the FACs psychometric structures of patients using handheld devices (i.e., smartphones and tablets) with those using personal computers (i.e., laptops and desktops). Measurement invariance results between the two device types are outlined in Table 4. All obtained goodness-of-fit indices met the criteria for configural invariance. Subsequently, the criteria for goodness-of-fit indices for metric invariance were satisfied when comparing handheld devices and computer users (Δ$\chi^{2}$(18) =14.62, *p* = 0.688, ΔRMSEA < 0.001, ΔCFI = 0.001, and ΔSRMR = 0.001). Additionally, the requirements for goodness-of-fit indices for the scalar invariance test were met (Δ$\chi^{2}$(18) = 20.96, *p* = 0.282, ΔRMSEA = −0.003, ΔCFI = −0.001, and ΔSRMR = 0.001). Finally, all criteria, except for the $\chi^{2}$ difference test for strict invariance indices, were fulfilled ($\chi^{2}$(20) = 116.98, *p* < 0.001, ΔRMSEA = 0.003, ΔCFI = −0.007, and ΔSRMR = 0.008), thereby establishing strict invariance.

In summary, these findings present evidence for configural, metric, scalar, and strict invariance of the two-factor model of FACs for men and women, and for the different device types used by our sample.

### 3.6. Associations between FACs Items and PHQ-8 and GAD-7 Items

Tables S1–S4 in the Supplementary Materials display correlations among FACs fatigue items, FACs altered cognition items, PHQ-8 items, and GAD-7 items. Using a Pearson *r* correlation > 0.70 as the criterion (i.e., more than 50% shared variance between the two items) for identifying highly correlated items, we found only two FACs fatigue items—Q1: “I felt fatigued” and Q20: “I had problems feeling energetic no matter if I slept or napped”—were highly correlated with the PHQ-8 item “Feeling tired or having little energy”, with *r*’s = 0.72 and 0.71, respectively. The majority of the FACs fatigue items had low to medium correlations with PHQ-8 items. With regard to the FACs altered cognition items, none of the correlations with PHQ-8 items were above 0.70. The highest correlations we observed were for Q5: “I had trouble concentrating” and Q9 “I had trouble focusing on things I wanted to do”, and Q18: “I had trouble paying attention” with one PHQ-8 item “Trouble concentrating on things, such as reading the newspaper or watching television” at 0.64, 0.64, and 0.65, respectively.

Regarding correlations with GAD-7 items, it is noteworthy that all FACs items exhibited coefficients below 0.60. This implies that the common variance shared between FACs items and GAD-7 items was less than 40%. In essence, while there were certain shared elements between FACs items and both PHQ-8 and GAD-7 items, these FACs items demonstrated distinctive characteristics that are independent of and non-redundant with these established measures of depression and generalized anxiety symptomology.

## 4. Discussion

The item analysis results indicate that all items of the FACs demonstrated appropriate utilization of the item response scales, with no evidence of floor or ceiling effects observed. Furthermore, the low percentage of missing data suggests minimal potential for respondents’ misunderstanding of item descriptions or respondent fatigue. The CFA results confirmed the intended two-factor (subscale) structure of the FACs, supporting the differentiation between brain fog and fatigue. These findings align with a previous study on the FACs [16]. Moreover, both item-scale correlations and reliability assessments (utilizing Cronbach’s alpha and omega coefficients) demonstrate high internal consistency within the subscales, indicating that composite item scores effectively capture brain fog and fatigue, respectively. Overall, the present study provides empirical support for the two-factor solution of the FACs and underscores its potential applicability in clinical research and practice with COVID-19 survivors, generally, and with patients with PASC. The concepts of brain fog and central fatigue, as measured by the FACs, appear to be best understood as separate, albeit related, entities that often co-occur across a variety of chronic health conditions.

We conducted analyses to examine the measurement invariance of the FACs’ factor structure across sex and device type categories, and the results consistently supported such invariance. Specifically, the instrument exhibited full invariance at the scalar level, indicating that the FACs maintains its item and factor integrity regardless of sex and the electronic device used for administration. Additionally, the brain fog and the fatigue scores can be meaningfully compared between males and females, as well as between personal computers and handheld devices. The absence of structural variation in FACs structure across sex and device types enhances confidence in the robustness of this measurement tool.

A close inspection of item correlations reveals the symptoms of brain fog and fatigue assessed by the FACs can be distinguished from the cardinal symptoms of major depression and generalized anxiety. Although depression severity is often associated with cognitive impairment among patients with PASC [51], research to date has yet to isolate the specific symptoms that appear on measures of depression and cognition that essentially assess the same complaints, potentially conflating the nature of the relationship of depression to brain fog and fatigue. The results of our study imply that differential diagnosis of co-occurring brain fog and fatigue among patients with possible depressive and anxiety disorders may be essential to clinical assessment and treatment planning with patients with PASC. Preliminary evidence indicates that brain fog and central fatigue among patients with TBI respond favorably to growth hormone therapy (although brain fog responds more readily than fatigue) [22]. Evidence-based psychological interventions should be considered for those patients who meet the criteria for depressive disorders (e.g., cognitive behavioral and behavioral activation therapies), and other interdisciplinary approaches (e.g., cognitive rehabilitation strategies and aerobic exercise) have been recommended to address the complex biopsychosocial issues that typify PASC [52]. Nevertheless, a thorough assessment is required to determine if, in fact, these patients experience cardinal symptoms required to meet the criteria to diagnose a depressive (or anxiety) disorder. Without an informed and expert assessment, our findings imply that symptoms of brain fog and central fatigue may be misconstrued as symptoms of mood disorders, in particular, which may then compromise clinical treatments.

The FACs provides clinicians with an efficient, valid, sensitive, and convenient tool that can expedite clinical assessment and monitoring of symptoms specific to brain fog and central fatigue associated with PASC. Its format can be easily adapted and configured for different handheld devices that can be used in the clinical setting (and in patient–clinician interactions). Respondents appear to easily understand the visual scales for the FACs items, and the psychometric properties reported here and in prior work [16] are consistent with the properties that would be observed with a traditional, paper administration. The results of the present study provide support for the need to develop improved and more precise measures of the behavioral and cognitive symptoms associated with long COVID [53]. Further research is needed to obtain more information about the discriminant and construct validity of the FACs among patients with PASC and other chronic health conditions in which the brain fog–fatigue symptom cluster is observed.

The rates of a probable major depressive disorder and a generalized anxiety disorder in the present study (20.9% and 24.5%, respectively) were lower than reported in a recent meta-analysis of these conditions post-COVID (depression, 45%; anxiety 47%) [10]. The PHQ-8 and the GAD-7 are rather conservative instruments, closely adhering to symptoms required for a diagnosis of a major depressive disorder and a generalized anxiety disorder. Lower rates may be expected when comparisons are made with instruments that do not strictly adhere to the criteria required to meet a diagnosis of a major depressive disorder or a generalized anxiety disorder. The rates of probable depression found in the present study are consistent with other work using the same cut-off scores to determine classification in an online survey (18.3% to 31% for major depression) [54], but lower than the rate observed in a multisite, clinic-based study of COVID-19 survivors (61%) [55]. These differences may reflect the unique characteristics of those who participate in online surveys post-COVID versus those who are seen in the clinic.

Women appear to be at greater risk than men for developing PASC and appear to experience more frequent and severe problems with perceived cognitive deficits, fatigue, depression, and anxiety [56,57,58]. The findings of the present study are consistent with this pattern but offer no insights into possible reasons for these differences. Consequently, further study of the possible risk factors that disproportionately affect women post-COVID is needed.

There are several limitations of the study that merit consideration. We relied on a sample of individuals who tested positive for COVID-19 from a single healthcare system, and who consented to complete an online survey that included several instruments. We do not know the degree to which this sample represents the larger number of COVID-19 survivors treated by this healthcare system, specifically, or from the larger geographic region. The relatively low number of participants in the sample who required hospitalization when treated for COVID-19 may suggest that the sample is not representative of those who experienced more severe cases of COVID-19. There are some data linking hospitalization to a greater risk of PASC, for example [59]. The lower rates of depression and anxiety determined by the cut-off scores on these respective instruments may be attributed, in part, to the time in which these data were collected (in 2021). In addition, clinically depressed and anxious individuals may have lacked motivation to participate in the study and complete the survey. We do not know how much time elapsed between the positive test for COVID-19 and the assessments completed in the online survey. It may be possible that symptoms of brain fog, fatigue, depression, and anxiety changed during that interval. Our reliance on self-report measures in the absence of corroborating clinical data (e.g., mental health diagnoses, PASC symptomology, and results from neuropsychological assessments) further limits the generalizability of the study. Studies of the FACs in combination with established neuropsychological instruments among COVID-19 survivors, particularly of those receiving outpatient services for PASC, are recommended.

## Figures and Tables

**Table 1:** Summary of participant characteristics.

Characteristics		
Age, *M*, *SD*	55.10	14.30
Sex, *N*, %		
Male	173	30.95
Female	380	67.98
Prefer not to answer or missing response	6	1.07
* Race/Ethnicity, *N*, %		
White	465	83.18
Asian	13	2.33
Hispanic	54	9.66
Black	40	7.16
Native Hawaiian or Pacific Islander	4	0.72
Other	9	1.61
Highest School Grade Completed, *N*, %		
9–11th grade	1	0.18
High school graduate/GED	38	6.80
Vocational/technical school	24	4.29
Associate degree/some college	126	22.54
Bachelor’s degree	168	30.05
Advanced degree	196	35.06
Other	1	0.18
Prefer not to answer or missing response	5	0.89
Yearly Household Income, *N*, %		
Less than USD 9999	9	1.61
USD 10,000 to USD 19,999	16	2.86
USD 20,000 to USD 29,999	23	4.11
USD 30,000 to USD 44,999	40	7.16
USD 45,000 to USD 59,999	51	9.12
USD 60,000 to USD 74,999	59	10.55
USD 75,000 to USD 99,999	79	14.13
USD 100,000 to USD 149,999	106	18.96
USD 150,000 or More	126	22.54
Prefer not to answer or missing response	50	8.94
* Chronic Health Condition, *N*, %		
Chronic Lung Disease (asthma/emphysema/COPD)	83	14.85
Diabetes Mellitus	81	14.49
Cardiovascular Disease (including high blood pressure, CHF)	176	31.48
Chronic Renal Disease	18	3.22
Liver Disease	13	2.33
Immunocompromised Condition	53	9.48
Neurologic/ neurodevelopmental/ intellectual disability	20	3.58
Traumatic Brain Injury	5	0.89
Spinal Cord Injury	10	1.79
Cancer	37	6.62
Other chronic diseases	70	12.52
Previously diagnosed with psychological condition, *N*, %		
No	367	65.65
Yes	171	30.59
Prefer not to answer or missing response	21	3.76

* Note: Respondents could select more than one response for these characteristics.

**Table 2:** Item analyses of the 20 FACS items.

Item	*n*	Min.	Max.	Mean	SD	Skewness	Item-Scale Correlations
**Fatigue Scale**							
Q1: I felt fatigued	555	0	100	52.30	31.80	−0.28	0.89
Q2: I felt alert *	539	0	100	38.33	24.37	0.38	0.68
Q6: I felt worn out	551	0	100	56.22	31.16	−0.38	0.91
Q7: I felt sluggish	553	0	100	51.67	30.97	−0.26	0.92
Q8: I felt run down	550	0	100	52.02	32.23	−0.27	0.92
Q10: I had the energy to do what I wanted to do *	552	0	100	47.55	27.35	−0.03	0.70
Q13: I had to force myself to get things done	552	0	100	48.39	32.22	−0.14	0.84
Q15: I felt tired	551	0	100	58.98	29.94	−0.51	0.88
Q17: I had to struggle to finish what I started to do	549	0	100	43.18	31.11	0.07	0.83
Q20: I had problems feeling energetic no matter if I slept or napped	550	0	100	50.05	33.13	−0.18	0.90
**Altered Cognition Scale**							
Q3: I lost track of what I was going to say	547	0	100	43.62	31.24	0.07	0.85
Q4: I was forgetful	546	0	100	45.12	30.91	0.04	0.89
Q5: I had trouble concentrating	547	0	100	44.86	32.03	0.06	0.91
Q9: I had trouble focusing on things I wanted to do	550	0	100	44.83	31.96	0.04	0.91
Q11: I was easily confused	548	0	100	29.11	28.44	0.79	0.86
Q12: I felt “spaced out” like I was in a fog	548	0	100	33.64	31.63	0.53	0.87
Q14: I was clear-headed *	544	0	100	40.93	28.04	0.26	0.78
Q16: I didn’t process things as quickly or accurately as I should have	548	0	100	41.95	30.91	0.14	0.89
Q18: I had trouble paying attention	553	0	100	40.86	30.30	0.17	0.91
Q19: It was hard for me to make up my mind and reach a decision	542	0	100	36.25	29.69	0.34	0.85

* Reverse-coded before conducting item analyses.

**Table 3:** Measurement invariance of the FACS by self-identified sex.

Model	$\chi^{2}$	df	RMSEA	ΔRMSEA	CFI	ΔCFI	SRMR	ΔSRMR
Configural	1307.365	328	0.098	-	0.922	-	0.060	-
Metric	1329.43	346	0.098	<0.001	0.922	<0.001	0.061	0.001
Scalar	1357.41	364	0.099	0.001	0.921	−0.001	0.062	0.001
Strict	1438.27	384	0.100	0.001	0.916	−0.005	0.064	0.002

Notes. $\chi^{2}$ = chi-square; df = degrees of freedom; CFI = comparative fit index; ΔCFI = delta (change in) CFI; RMSEA = root mean square error of approximation; ΔRMSEA = delta (change in) RMSEA; SRMR = standardized root mean square residual (SRMR); ΔSRMR = delta (change in) SRMR.

**Table 4:** Measurement invariance of the FACS between handheld and personal computer devices.

Model	$\chi^{2}$	df	RMSEA	ΔRMSEA	CFI	ΔCFI	SRMR	ΔSRMR
**Configural**	1233.178	328	0.099	-	0.929	-	0.057	-
**Metric**	1247.794	346	0.099	<0.001	0.930	0.001	0.058	0.001
**Scalar**	1268.750	364	0.096	−0.003	0.929	−0.001	0.059	0.001
**Strict**	1385.726	384	0.099	0.003	0.922	−0.007	0.067	0.008

Notes. $\chi^{2}$ = chi-square; df = degrees of freedom; CFI = comparative fit index; ΔCFI = delta (change in) CFI; RMSEA = root mean square error of approximation; ΔRMSEA = delta (change in) RMSEA; SRMR = standardized root mean square residual (SRMR); ΔSRMR = delta (change in) SRMR.

## Data Availability

The data set from which the survey on which the present study is based is not publicly available due to the inclusion of private health information. The data analyzed in this report are available from the first author upon reasonable request.

## Supplementary Materials

The following supporting information can be downloaded at: https://www.mdpi.com/article/10.3390/jcm13082186/s1, Table S1, Correlations between the FACs Fatigue Scale items and the PHQ-8 items; Table S2, Correlations between the FACs Fatigue Scale items and the GAD-7 items; Table S3, Correlations between the FACs Altered Cognition Scale items and the PHQ-8 items; Table S4, Correlations between the FACs Altered Cognition Scale items and the GAD-7 items.
