# Supplementary material for: The Fatigue and Altered Cognition Scale among SARS-CoV-2 Survivors: Psychometric Properties and Item Correlations with Depression and Anxiety Symptoms
Source: J Clin Med. 2024 Apr 10;13(8):2186. doi: 10.3390/jcm13082186 (PMC11050894; doi:10.3390/jcm13082186)
Supplement: Supplementary file 1 [file jcm-13-02186-s001.zip › jcm-2902220-supplementary.pdf]

Journal of Clinical Medicine

MDPI

Table S1. Correlations between the FACs Fatigue Scale items and the PHQ-8 items.

	PHQ-8_1	PHQ-8_2	PHQ-8_3	PHQ-8_4	PHQ-8_5	PHQ-8_6	PHQ-8_7	PHQ-8_8
**Fatigue Scale**								
Q1: I felt fatigued	.557	.532	.511	**.721**	.512	.416	.485	.363
Q2: I felt alert*	.477	.443	.382	.554	.370	.375	.495	.317
Q6: I felt worn out	.522	.508	.464	.664	.463	.395	.478	.299
Q7: I felt sluggish	.546	.506	.495	.679	.460	.380	.502	.337
Q8: I felt run down	.547	.518	.459	.673	.473	.400	.512	.321
Q10: I had the energy to do what I wanted to do*	.467	.410	.382	.519	.397	.345	.410	.205
Q13: I had to force myself to get things done	.545	.513	.410	.584	.472	.421	.529	.346
Q15: I felt tired	.479	.467	.469	.644	.430	.370	.431	.263
Q17: I had to struggle to finish what I started to do	.544	.472	.439	.564	.465	.427	.585	.376
Q20: I had problems feeling energetic no matter if I slept or napped	.583	.495	.486	**.708**	.506	.415	.527	.354

Table S2. Correlations between the FACs Fatigue Scale items and the GAD-7 items.

	GAD-7_1	GAD-7_2	GAD-7_3	GAD-7_4	GAD-7_5	GAD-7_6	GAD-7_7
**Fatigue Scale**							
Q1: I felt fatigued	.539	.505	.536	.558	.412	.518	.487
Q2: I felt alert*	.427	.415	.401	.457	.336	.452	.418
Q6: I felt worn out	.534	.495	.523	.531	.399	.508	.465
Q7: I felt sluggish	.523	.498	.513	.530	.431	.530	.483
Q8: I felt run down	.525	.476	.505	.526	.404	.523	.460
Q10: I had the energy to do what I wanted to do*	.387	.407	.408	.419	.303	.399	.349
Q13: I had to force myself to get things done	.568	.520	.534	.571	.453	.545	.496
Q15: I felt tired	.489	.452	.485	.499	.346	.479	.433
Q17: I had to struggle to finish what I started to do	.531	.495	.531	.543	.455	.518	.485
Q20: I had problems feeling energetic no matter if I slept or napped	.541	.492	.527	.548	.429	.528	.468

Table S3. Correlations between the FACs Altered Cognition Scale items and the PHQ-8 items.

	PHQ-8_1	PHQ-8_2	PHQ-8_3	PHQ-8_4	PHQ-8_5	PHQ-8_6	PHQ-8_7	PHQ-8_8
**Altered Cognition Scale**								
Q3: I lost track of what I was going to say	.430	.455	.403	.456	.398	.346	.493	.306
Q4: I was forgetful	.456	.487	.411	.484	.358	.389	.530	.354
Q5: I had trouble concentrating	.512	.494	.435	.553	.411	.394	.640	.389
Q9: I had trouble focusing on things I wanted to do	.555	.517	.458	.580	.479	.393	.639	.381
Q11: I was easily confused	.499	.437	.400	.498	.378	.368	.594	.461
Q12: I felt “spaced out” like I was in a fog	.501	.487	.418	.523	.392	.382	.583	.442
Q14: I was clear-headed*	.514	.500	.401	.540	.413	.425	.545	.405
Q16: I didn’t process things as quickly or accurately as I should have	.539	.530	.446	.526	.439	.441	.590	.389
Q18: I had trouble paying attention	.520	.490	.442	.526	.454	.414	.651	.413
Q19: It was hard for me to make up my mind and reach a decision	.482	.461	.382	.485	.411	.416	.569	.360

Table S4. Correlations between the FACs Altered Cognition Scale items and the GAD-7 items.

	GAD-7_1	GAD-7_2	GAD-7_3	GAD-7_4	GAD-7_5	GAD-7_6	GAD-7_7
**Altered Cognition Scale**							
Q3: I lost track of what I was going to say	.492	.442	.454	.444	.355	.489	.409
Q4: I was forgetful	.506	.457	.483	.481	.396	.500	.456
Q5: I had trouble concentrating	.550	.520	.545	.553	.445	.561	.491
Q9: I had trouble focusing on things I wanted to do	.565	.535	.561	.563	.462	.566	.502
Q11: I was easily confused	.498	.463	.488	.487	.465	.520	.468
Q12: I felt “spaced out” like I was in a fog	.532	.503	.535	.529	.480	.541	.493
Q14: I was clear-headed*	.487	.460	.466	.492	.444	.506	.450
Q16: I didn’t process things as quickly or accurately as I should have	.538	.508	.522	.529	.446	.530	.501
Q18: I had trouble paying attention	.538	.491	.530	.555	.499	.548	.493
Q19: It was hard for me to make up my mind and reach a decision	.528	.510	.524	.507	.444	.537	.502
